# Transport of Peripartum Patients for Medical Management: Predictors of Any Intervention During Transport

**DOI:** 10.7759/cureus.31102

**Published:** 2022-11-04

**Authors:** Quincy K Tran, Grace Hollis, Richa Beher, Maie Abdel-Wahab, Gaurika Mester, Saman Tanveer, Katherine Jones, Allison Lankford, Ann Matta, Rose Chasm

**Affiliations:** 1 Department of Critical Care, University of Maryland School of Medicine, Baltimore, USA; 2 The Research Associate Program, Department of Emergency Medicine, University of Maryland School of Medicine, Baltimore, USA; 3 Department of Critical Care Medicine, Miller School of Medicine, Miami, USA; 4 Department of Critical Care Medicine, R Adams Cowley Shock Trauma Center, University of Maryland School of Medicine, Baltimore, USA; 5 Department of Obstetrics, Gynecology and Reproductive Sciences, University of Maryland School of Medicine, Baltimore, USA; 6 Department of Emergency Medicine, R Adams Cowley Shock Trauma Center, University of Maryland School of Medicine, Baltimore, USA; 7 Department of Emergency Medicine, University of Maryland School of Medicine, Baltimore, USA

**Keywords:** interhospital transport, emergency medicine, medical management, obstetric, peripartum

## Abstract

Background

When obstetric patients present to a hospital without obstetric consultants for medical care, they are often transferred for a higher level of care. Factors associated with patient care during transport between hospitals are unknown. Our study investigated factors associated with care intensity.

Methods

We retrospectively reviewed charts from peripartum adult patients who were transported to our academic quaternary center between January 1, 2012, and April 19, 2020. We excluded patients transported for gynecologic or fetus-related complications. We defined any transport interventions as any ventilator management, any titration of continuous infusions, and any continuation or initiation of medication during transport.

Results

We included 126 patients, and the mean age (SD) was 29 (7) years. There were 87 (695) antepartum patients, with nine (7%) requiring mechanical ventilation. Of the patients, 98 (78%) received at least one intervention during transport. Transport time (OR: 1.03, 95% CI: 1.001-1.06, P = 0.040), preeclampsia (OR: 3.8, 95% CI: 1.1-12.7, P = 0.030), and any obstetric hemorrhage (OR: 8.3, 95% CI: 1.03-68, P = 0.047), either antepartum or postpartum, were associated with higher likelihood of receiving any intervention.

Conclusions

Peripartum patients often received interventions during transport. Preeclampsia and hemorrhage were associated with the likelihood of interventions. Transport clinicians should be prepared when transporting these patients. Further studies are needed to confirm our observations.

## Introduction

Interhospital transfers (IHT) have become more common in recent years, especially with the advancement of specialty services. With this recognition, organizations responsible for transporting these patients have developed protocols and guidelines to avoid untoward complications during transport. Although the transport of patients between hospitals is common, transport of pregnant patients is less common, resulting in limited data. The 2014-2016 National Emergency Medical Services (EMS) database showed that only 0.6% of transfers involved pregnant patients [1].

Few studies have investigated the IHT care for pregnant patients between hospitals [2-6]. However, all these studies only reported the epidemiology of what occurred during transport, or they only involved antepartum patients. Furthermore, these previous studies [2-6] did not report patient-level data such as blood pressure measurements, types of medical interventions during transport, and care intensity. The lack of data regarding types of medical interventions and level of care intensity during the IHT process poses challenges for transport clinicians because these obstetric patients, although a small percentage of the total IHT patient population, involve complex physiology and special knowledge for effective management. A previous study by Jones et al. [7], which surveyed 133 air medical programs in the United States, demonstrated that the greatest concerns for medical personnel were in-flight delivery, inadequate fetal monitoring, and inexperience with care for obstetric patients, respectively.

The transport team’s management during transport is a vital component of patient care. Higher care intensity during transport between hospitals was associated with improved patients’ short-term outcomes at the accepting hospitals [8]. Furthermore, being able to predict the level of care intensity or what type of interventions obstetric patients may need during the IHT process would potentially provide transport clinicians with valuable information to improve patients’ care and outcomes. Knowing what types of interventions peripartum patients who are transported for medical reasons would require also provide the transport clinicians with the comfort and knowledge about how to prepare for these patients during the transport process.

This study aimed to investigate the care intensity provided by transport clinicians to a special group of obstetric patients who were transported between hospitals for the management of non-delivery-related medical conditions.

## Materials and methods

Study settings and patient selection

This was a retrospective, single-center study. The academic quaternary medical center, University of Maryland Medical Center (UMMC), which accepts patients from throughout the state of Maryland and the nearby region, has an obstetrics and gynecology (OBGYN) specialty that provides consultation and admission for eligible patients around the clock. When referring physicians from other hospitals need to transfer patients to the OBGYN service, they contact the specialized transfer center, ExpressCare, at the UMMC, which facilitates the interhospital transfer of patients. The UMMC received 50,599 transfer requests for all pediatric, adult, and adult trauma patients between the calendar years 2014 and 2018, with an acceptance rate of approximately 91%. A list of all patients who are transferred to UMMC and the list of the primary accepting services for those patients are maintained by ExpressCare.

The analysis included all patients who were transferred from other hospitals to UMMC between January 1, 2012, and April 19, 2020, and who were admitted to the OBGYN service as the primary accepting service. Only patients who were transferred for medical management were included in the study. To identify qualifying diagnoses and eligible patients, a critical care fellowship-trained obstetrician initially screened patients’ diagnoses at the time of transfer request. Indications for transport due to the need for medical management were defined as eclampsia, preeclampsia, and other pulmonary (asthma, etc.) or cardiovascular (heart failure) conditions. Non-obstetric infection was defined as urinary tract infection, pneumonia, cholecystitis, appendicitis, etc., in contrast to obstetric infection such as chorioamnionitis. Any obstetric hemorrhage was considered medical management as it would initially be managed medically. Non-medical indications were defined as in a previous study (preterm labor, preterm premature rupture of membranes (PPROM) or premature rupture of membranes (PROM), and threatened abortion) [2]. Since this study focused on transport management, only the initial diagnoses at the time of transfer request were considered, and not the diagnoses upon hospital discharge.

Patients who did not have transport clinicians’ documentation, patients who were transported for management of gynecologic conditions (pelvic mass, ovarian torsions, etc.), or patients who were not accepted to the OBGYN service as the primary accepting service were excluded. This study was reviewed by the Institutional Review Board and formal consent was exempted.

Interventions during transport

All interventions rendered by transport clinicians during transport between hospitals were considered clinicians’ interventions. Interventions were further categorized as (a) administration of new medication, including starting intravenous crystalloids, (b) continuing or titrating medications that were started by the referring clinicians, and (c) any change of mechanical ventilation or oxygen support during transport. However, crystalloid infusion with the purpose to keep veins open (KVO) or providing carrier solutions for other medications or blood products was not considered an intervention.

Outcome

The primary outcome was the percentage of patients who received any interventions during transport. We hypothesized that at least 50% of the patients would receive any intervention, as a previous study suggested that 48% of patients who were transported from another hospital to a resuscitation unit received any interventions [9]. The secondary outcome was any clinical predictors for interventions during transport.

Sample size analysis

In this retrospective study, a priori sample size analysis was not performed as there is no prior information about care intensity in this particular patient population. Since the study population was unique, the plan was to include as many patients as possible during the study period.

Data collection and management

The principal investigator trained other research team members, who were blinded to the study’s hypothesis, in data extraction. To reduce bias and increase accuracy, investigators were trained by repeatedly collecting data from sets of 10 patients’ charts until the interrater's agreement reached 90%. Additionally, one investigator independently checked 20% of the data of other investigators. Any discrepancies were rechecked and the results were adjudicated among investigators with the principal investigator serving as the tie-breaker. Data were extracted into a standardized Excel spreadsheet (Microsoft Corporation, Redmond, WA). Demographic and clinical data were collected from transport clinicians’ documentation that was included with patients’ paper charts upon arrival at UMMC. Demographic data included the age of mothers, gestational age (if not postpartum), types of transport (air or ground, academic affiliation), and level of transport (nursing or paramedic). Clinical data included diagnoses at transfer request, blood pressure, heart rate, and mechanical ventilation. Outcomes of mothers and fetuses were collected from the medical center’s electronic medical records.

Data analysis

Descriptive analysis with a mean (standard deviation (SD)) or median (interquartile range (IQR)) was used to demonstrate the patient’s demographic and clinical information. Continuous data were compared using the Student's t-test or the Mann-Whitney U test as appropriate, and categorical data were compared with Pearson’s chi-square or the Fisher's exact test as appropriate.

The outcome was dichotomized as no intervention vs. any intervention. Subsequently, multivariable logistic regression was performed to assess the association between demographic and clinical predictors with the outcome of any intervention during transport. All independent variables were selected a priori and were listed in Table 1. To optimize the goodness-of-fit of the regression, only factors that had a strong association with the outcome during bivariate analyses were included, i.e., only factors with p-values for the t-tests or Mann-Whitney U tests ≤ 0.10 [10].^ ^The goodness-of-fit of the models was assessed by the Hosmer-Lemeshow test. A model with a Hosmer-Lemeshow test’s p-value > 0.05 was considered as having a good fit of independent variables. Any multicollinearity was assessed by the variance inflation factor (VIF). Any variable with VIF > 5 was considered as having multicollinearity and would be removed from the regression. Statistical analyses were performed via Minitab version 19.0 (Minitab Inc., State College, PA). Any two-tailed p-value < 0.05 was considered significant.

**Table 1:** List of independent continuous and categorical variables * Other diagnoses include thromboembolic disease, placental abnormalities, diabetic ketoacidosis, hypertension, hematologic disorder, and seizure not otherwise specified. ALS, advanced life support; BLS, basic life support; ED, emergency department; ICU, intensive care unit; L&D, labor and delivery unit; Med Ward, general medical ward.

Continuous variables
Age, years
Initial and arrival systolic blood pressure (SBP)
Initial and arrival shock index (SI)
Length of transport
Gravida and para
Categorical variables
Antepartum (Yes = 1, No = 0)
Sending unit type (Yes = 1, No = 0): ED, ICU, L&D, Med Ward
Academic-affiliated hospital (Yes = 1, No = 0)
Transport type (Air = 1, Ground = 0)
Transport level (Yes = 1, No = 0): Nurse, ALS, BLS
Mechanical ventilation (Yes = 1, No = 0)
Top 10 diagnoses (Yes = 1, No = 0)
Cardiac problem
Cardiomyopathy, pulmonary hypertension, structural heart problem, arrhythmia
Eclampsia
Non-obstetric infection
Obstetric hemorrhage
Preeclampsia
Respiratory
Surgical
Trauma
Other*

## Results

Patient demographic

**Figure 1:**
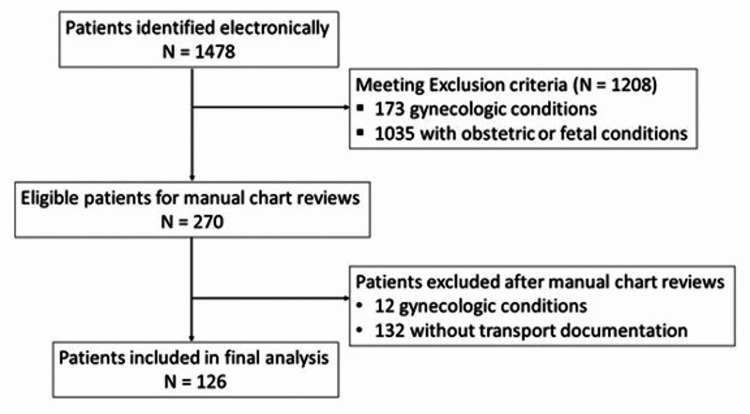
Flow diagram of patient selection

During the study period, the obstetric service accepted for transfer of 1478 peripartum patients for all obstetric-related conditions (Figure 1). However, 126 patients who met eligibility criteria were included for analysis (Figure 1).

The mean (±SD) age was 29 (±7) years. A total of 95 (75%) patients were antepartum with a mean (±SD) gestational age of 33 (±12) weeks. Half of the patients were transferred from labor & delivery units (63/126, 50%), another third (42/126, 33%) were transferred from emergency departments, and the remaining patients came from intensive care units (18/126, 14%) and medical wards (3/126, 2%). The majority of patients (111/26, 88%) were transported via ground for a mean of 36 (±20) minutes (Table 2). The most common diagnoses were preeclampsia with 37 (37/126, 29%) patients. All diagnoses at transport were listed in Table 3.

**Table 2:** Demographic characteristics of interhospital transferred peripartum patients * We only reported the top five diagnoses here. N, number of patients; IQR, interquartile range; SD, standard deviation; P, p-value.

Variables	All patients (N = 126)	Patients receiving no interventions (N = 28, 22%)	Patients receiving one or more interventions (N = 98, 78%)	P
Age, years, mean (SD)	29 (7)	29 (7)	29 (7)	0.90
Antepartum, N (%)	95 (75)	25 (89)	70 (71)	0.05
Gestational age, weeks, mean (SD)	33 (12)	28 (11)	34 (11)	0.01
Gravida status, median (IQR)	3 (1-4)	2 (2-4)	3 (1-4)	0.90
Para status, median (IQR)	1 (0-2)	1 (1-2)	1 (0-2)	0.60
Referring unit type, N (%)				
Emergency department	42 (33)	13 (46)	29 (30)	0.10
Intensive care unit	18 (14)	1 (4)	17 (17)	0.07
Labor and delivery	63 (50)	13 (46)	50 (51)	0.70
Medical ward	3 (2)	1 (4)	2 (2)	0.60
Type of transport, N (%)				
Ground	111 (88)	27 (96)	84 (86)	0.10
Air	15 (12)	1 (4)	14 (14)	0.10
Academically affiliated	117 (93)	26 (93)	91 (93)	0.99
Transport, N (%)				
Transport level, N (%)				
Registered nurse	115 (91)	27 (96)	88 (90)	0.30
Advanced life support	11 (9)	1 (4)	10 (10)	0.30
Diagnoses at time of transfer, N (%)*				
Preeclampsia	38 (29)	4 (14)	33 (34)	0.047
Eclampsia	13 (10)	1 (4)	12 (12)	0.20
Any obstetric hemorrhage	23 (18)	1 (4)	22 (22)	0.02
Non-obstetric infection	7 (6)	6 (21)	1 (1)	0.01
Respiratory distress or failure	7 (6)	2 (7)	5 (5)	0.70

**Table 3:** List of diagnoses at the time of transfer request NOS, not otherwise specified; PH, pulmonary hypertension; N, number of patients.

Diagnoses	N (%)
Hypertension	2 (2)
Preeclampsia	37 (29)
Eclampsia	13 (10)
Obstetric hemorrhage	23 (18)
Hematologic disease	4 (3)
Diabetic ketoacidosis	2 (2)
Surgical disease	5 (4)
Cardiomyopathy, PH, structural heart problem, arrhythmia	4 (3)
Thromboembolic disease	5 (4)
Placental abnormalities	4 (3)
Drug, alcohol abuse	2 (2)
Non-obstetric infection	8 (6)
Respiratory compromise, failure	7 (6)
Seizure NOS	2 (2)
Sepsis	2 (2)
Trauma	2 (2)
Other	4 (3)

Patients’ clinical characteristics

As shown in Table 4, among 126 patients being transported, 28 (28/126, 22%) did not receive any intervention while 98 (98/126, 78%) patients received at least one intervention(s). The majority of patients (120/126, 95%) were discharged home directly from the hospital while six patients (6/126, 5%) were discharged to a rehabilitation facility. No patients in this study died at the hospital.

**Table 4:** Clinical characteristics and outcomes of interhospital transferred peripartum patients SD, standard deviation; N, number of patients; BP, blood pressure; mmHg, millimeters of mercury; NICU, neonatal intensive care unit; P, p-value; N/A, not applicable.

Variables	All patients (N = 126, 100%)	No interventions (N = 28, 22%)	Any intervention (N = 98, 78%)	P
Initial systolic BP, mmHg, mean (SD)	132 (25)	131 (27)	132 (25)	0.80
Initial shock index, mean (SD)	0.77 (0.23)	0.71 (0.17)	0.78 (0.26)	0.04
Arrival systolic BP, mmHg, mean (SD)	131 (24)	130 (29)	131 (23)	0.90
Arrival shock index, mean (SD)	0.77 (0.22)	0.70 (0.17)	0.79 (0.24)	0.01
Requiring mechanical ventilation, N (%)	14 (11)	0 (0)	14 (14)	N/A
Length of transport, minutes, mean (SD)	36 (20)	27 (14)	39 (21)	0.01
Mother’s hospital disposition, N (%)				
Alive and discharged home	120 (95)	27 (96)	93 (95)	0.10
Rehabilitation	6 (5)	1 (4)	5 (5)	0.10
Fetal discharge status, N (%)				
Alive or NICU	47 (37)	16 (57)	31 (32)	0.01
Unborn	37 (29)	7 (25)	30 (31)	0.30
Dead	11 (9)	1 (4)	10 (10)	0.99
Other (postpartum)	31 (25)	4 (14)	27 (28)	0.20

Interventions During Transport

The median (IQR) of intervention per patient was 1 [1,2]. There were 35 (35/126, 28%) patients who received one intervention, 42 (42/126, 33%) patients who received two interventions, and 21 (21/126, 17%) patients who received three or more interventions. The most common type of intervention for pregnant patients during transport was continuing intravenous crystalloids (72/126, 57%) while initiating new intravenous crystalloids in nine (9/126, 7%) patients. Transport clinicians initiated new sedatives in six (6/126, 5%) patients. These data are shown below in Table 5.

**Table 5:** Care intensity during transport of interhospital transferred peripartum patients N, number of patients; P, p-value; FiO2, fraction of inspired oxygen; N/A, not applicable as the statistical analysis was not performed.

Intervention	All patients (N = 126, 100%)	Low intensity of care (1 intervention, N = 35)	Medium intensity of care (2 interventions, N = 42)	High intensity of care (3 or more interventions, N = 21)	P (high vs. medium)
Total interventions per patient, median (IQR)	1 (1-2)	1 (1-1)	2 (2-2)	4 (3-6)	0.01
Continued medications, N (%)					
Crystalloids	72 (57)	23 (66)	35 (83)	14 (67)	0.16
Blood products	7 (6)	0 (0)	2 (5)	5 (24)	N/A
Antibiotics	7 (6)	4 (11)	1 (2)	2 (10)	0.27
Anti-hypertensives	3 (2)	0 (0)	0 (0)	3 (14)	N/A
Titration of medications, N (%)					
Sedatives	3 (2)	0 (0)	0 (0)	3 (14)	N/A
Crystalloids	3 (2)	0 (0)	3 (7)	0 (0)	N/A
Anti-hypertensives	3 (2)	0 (0)	0 (0)	3 (14)	N/A
New medications, N (%)					
Crystalloids	9 (7)	3 (9)	5 (12)	1 (5)	0.64
Sedatives	6 (5)	0 (0)	2 (5)	4 (19)	N/A
Blood products	2 (2)	1 (3)	0 (0)	1 (5)	N/A
Mechanical ventilation, N (%)	14 (11)	1 (3)	3 (7)	10 (48)	0.01
Any ventilation change, N (%)	5 (4)	0 (0)	0 (0)	5 (24)	N/A
Types of ventilation change, N (%)					
FiO2	2 (2)	0 (0)	0 (0)	2 (10)	N/A
Respiratory rate	2 (2)	0 (0)	0 (0)	2 (10)	N/A

Only 14 (14/126, 11%) pregnant patients required invasive mechanical ventilation, without requiring frequent ventilation changes. There were only two (2/126, 2%) adjustments in the fraction of inspired oxygen (FiO2) and two (2/126, 2%) adjustments in respiratory rates during transport. These data are shown in Table 5.

Factors Associated With Interventions

Multivariable logistic regression containing independent variables with a strong association with outcome showed four factors with statistically significant association with the outcome of any intervention as seen below in Table 6. Transport time (OR: 1.03, 95% CI: 1.001-1.06, P = 0.040), preeclampsia (OR: 3.8, 95% CI: 1.14-12.7, P = 0.030), and obstetric hemorrhage (OR: 8.3, 95% CI: 1.03-68, P = 0.047) were associated with higher likelihood of receiving any intervention during transport. Conversely, non-obstetric infection (OR: 0.1, 95% CI: 0.01-0.8, P = 0.035) was associated with a lower likelihood of requiring intervention during transport.

**Table 6:** Results from multivariable logistic regression

Variables	Odds ratio	95% CI	P
Length of transport – each minute	1.03	1.001-1.06	0.040
Preeclampsia	3.8	1.14-12.7	0.030
Any obstetric hemorrhage	8.3	1.03-68	0.047
Non-obstetric infection	0.1	0.01-0.8	0.035

The regression showed a good fit of the variables, as the Hosmer-Lemeshow test’s p-value was 0.63. There was no multicollinearity among the variables because all factors had good VIF between 1.02 and 1.05.

## Discussion

This study showed that among the IHT peripartum patients who were transported for further management of medical conditions, 28% (35/125) of the patients received one intervention, while 33% (42/126 patients) and 17% (21/126 patients) received three or more interventions during transport, respectively. Furthermore, we identified three patient-specific conditions that were associated with any intervention. In this study, peripartum patients were commonly transferred for preeclamptic, eclamptic, and hemorrhagic conditions. Furthermore, titrating anti-hypertensive infusions and managing sedatives were associated with higher care intensity among this patient population. Both transport teams and referring clinicians should pay more attention to patients’ hemodynamic goals and sedative regiments among mechanically ventilated peripartum patients.

Among peripartum patients who were transported for medical care, this study highlighted the types of interventions for these patients. The percentage of peripartum patients in this study who received any interventions was higher than the 48% of patients who were transported to a resuscitation unit in a previous study [9]. Additionally, a lower percentage of obstetric patients required invasive mechanical ventilation and vasopressor support compared to the general critically ill patients being transported to a resuscitation unit as reported previously [9]. Furthermore, there were fewer ventilator changes in these peripartum patients, when compared to the general population of critically ill patients. This was likely because not all pregnant patients within this study were critically ill, yet transport clinicians would still provide higher care intensity to support these high-risk patients. The lower rates of ventilator change also suggested that peripartum patients in this study were overall healthier and had less issues with oxygenation or ventilation.

There is scant information in the literature regarding care intensity between air versus ground transport. The result from this study’s multivariable logistic regression did not identify that air transport was an independent factor that was associated with any interventions. The finding of this study was different from a previous study. A survey study from 852 patients being transported to a resuscitation unit showed that air transport clinicians were associated with higher care intensity [9]. In contrast, another retrospective study involving 200 patients with acute aortic diseases suggested that ground transport had similar levels of care during transport between hospitals [11]. Additionally, air transport was reported to cost eight times more than ground transport for patients with aortic dissection but the outcome between air transport and ground transport for these patients was similar [12-14]. As a result, the benefit of air transport in certain medical conditions, besides shorter traveling time, is not clearly delineated. The general outcome from the peripartum patients in this study was impressive overall, as there was no mortality for the mothers, and the majority of the patients were discharged home directly after specialized management at UMMC. As a result, referring physicians and transport clinicians should consider ground transport when the patients are adequately resuscitated unless the fetus’ health status is significantly threatened.

Fetal heart monitoring is an important part of the medical care of antepartum patients. Although Elliott et al. in 1987 reported that fetal heart rate monitoring was feasible, as the authors reported that 40 (70%) of pregnant patients being transported had fetal heart rate monitoring [15]. However, there was no record of fetal heart rate monitoring in the patient population studied. This could be due to the lack of equipment and lack of familiarity with the technology or knowledge to interpret tracings [16]. New technology could improve the convenience of fetal cardiotocography in transport using 5G transmission of fetal information to transport clinicians and receiving hospitals [17], thus improving patient care during transport.

Implications for future research

Further study involving a larger pregnant and postpartum patient population is necessary to confirm the observation that obstetric patients who were transferred for medical care had positive outcomes. Although up to 78% of patients in the patient population received any intervention, it is unclear whether those interventions or level of care intensity would be associated with a change in patient outcomes. Future studies should investigate the effects of transport clinicians’ actions and patient outcomes as results from such studies would provide transport clinicians with evidence-based recommendations for quality practice improvement, along with more educational opportunities. Furthermore, it would be important to investigate the feasibility of the technology for fetal heart rate monitoring during transport. Having this capability could improve patient care while reducing cost, as it is possible that referring physicians utilize air transport to reduce the amount of time without fetal heart rate monitoring.

Limitations

This study had several limitations. First, fetal heart monitoring is an important part of the medical care of antepartum patients. Although Elliott et al. in 1987 reported that fetal heart rate monitoring was feasible, as the authors reported that 40 (70%) of pregnant patients being transported had fetal heart rate monitoring [15]. However, there was no record of fetal heart rate monitoring in the patient population. This could be due to the lack of equipment and lack of familiarity with the technology or knowledge to interpret tracings [17]. New technology could improve the convenience of fetal cardiotocography in transport using 5G transmission of fetal information to transport clinicians and receiving hospitals, thus improving patient care during transport [17].

This study also has limited generalizability because it is based on the experience of a single institution in a metropolitan urban setting and a very small sample size. Results may not be generalizable to a less densely populated rural setting where transport times could be longer. Lack of documentation from transport teams further reduced the number of eligible patients and it was possible that transport clinicians only included documents with any interventions. To confirm that the study results were not affected by the number of exclusions, we considered a hypothetical scenario where each of the missing patients would have received the same number of interventions according to their diagnoses (Table 7). The resulting multivariable regression showed similar results to the primary analysis (Table 8). Adverse events during transport were not assessed, as a previous study suggested a low prevalence of adverse events among all transported pregnant women [2], and were mostly pregnancy-related events such as increased contractions [3].^ ^We did not have data to assess patients’ disease severity during transport. However, based on the low prevalence of mechanical ventilation or vasopressor requirement, the number of patients with high disease severity is likely to be low. Additionally, we only had outcomes of patients or fetuses at hospital discharges as all of the patients were transferred from other hospitals and we did not have access to their records after they were discharged. Nonetheless, this exploratory study had several strengths. To our knowledge, this is the first study that specifically investigates the interventions for a group of peripartum patients during interhospital transfer. We were also able to analyze and highlight the care intensity that was rendered by transport clinicians in this specialized group of patients.

**Table 7:** Comparison of demographic and clinical outcomes of included patients and missing patients N, number of patients; P, p-value; IQR, interquartile range; N/A, not applicable as the statistical analysis was not performed; SD, standard deviation.

Variable	All patients (included patients & missing patients) (N = 258)	Included patients (N = 126)	Missing patients (N = 132)	P
Age, mean (SD)	29 (7)	29 (7)	29 (7)	0.70
Antepartum, N (%)	204 (79)	95 (75)	109 (83)	0.20
Gravida, median (IQR)	2 (1-4)	3 (1-4)	3 (1-4)	0.99
Para, median (IQR)	0 (0-2)	1 (0-2)	1 (0-2)	0.99
Maternal discharge status, N (%)				
Alive, home	248 (96)	119 (98)	129 (97)	0.17
Alive, acute care or rehab	9 (3)	7 (2)	2 (2)	0.08
Dead	1 (1)	0 (0)	1 (1)	0.97
Fetal discharge status, N (%)				
Alive	73 (28)	38 (30)	35 (27)	0.50
Unborn	121 (47)	48 (38)	73 (55)	0.006
Dead	15 (6)	11 (9)	4 (3)	0.051
N/A (postpartum)	49 (19)	29 (23)	20 (15)	0.10
Diagnosis, N (%)				
Non-obstetric infection	19 (7)	8 (6)	11 (8)	0.50
Preeclampsia	68 (26)	37 (30)	31 (24)	0.30
Eclampsia	22 (9)	13 (10)	9 (7)	0.30
Hematologic disorder	11 (4)	4 (3)	7 (5)	0.40
Obstetric hemorrhage	32 (13)	23 (18)	9 (7)	0.005
Cardiac problem	11 (4)	4 (3)	7 (5)	0.40
Respiratory problem	12 (5)	7 (6)	5 (4)	0.50
Surgical problem	16 (6)	5 (4)	11 (8)	0.10
Thromboembolic disease	10 (4)	5 (4)	5 (4)	0.99
Other	57 (22)	20 (16)	37 (28)	0.019
Total intervention per patient, N, median (IQR)	1 (1-3)	1 (1-2)	1 (1-3)	0.98
Receiving any intervention, N (%)	219 (85)	98 (78)	121 (92)	0.005

**Table 8:** Results from the multivariable logistic regression

Variables	Odds ratio	95% CI	P
Preeclampsia	7.9	2.6-24	0.001
Eclampsia	8.3	1.03-50+	0.047
Obstetric hemorrhage	12	1.6-50+	0.015

## Conclusions

Up to 78% of obstetric patients who were transferred between hospitals for medical management required acute intervention during transport. Titrating anti-hypertensive infusions and managing sedative regimens were associated with higher care intensity among these patients. Therefore, both referring clinicians and transport teams should pay further attention to patients’ hemodynamic goals and sedation. Further studies are necessary to confirm the observations made in this study.

## References

[1] (2017). National EMS Database. NEMSIS research data set. Emergency.

[2] Nawrocki PS, Levy M, Tang N, Trautman S, Margolis A (2019). Interfacility transport of the pregnant patient: a 5-year retrospective review of a single critical care transport program. Prehosp Emerg Care.

[3] O'Brien DJ, Hooker EA, Hignite J, Maughan E (2004). Long-distance fixed-wing transport of obstetrical patients. South Med J.

[4] Jony L, Baskett TF (2007). Emergency air transport of obstetric patients. J Obstet Gynaecol Can.

[5] Akl N, Coghlan EA, Nathan EA, Langford SA, Newnham JP (2012). Aeromedical transfer of women at risk of preterm delivery in remote and rural Western Australia: why are there no births in flight?. Aust N Z J Obstet Gynaecol.

[6] Rzońca E, Bień A, Wejnarski A, Gotlib J, Gałązkowski R (2021). Polish medical air rescue interventions concerning pregnant women in Poland: a 10-year retrospective analysis. Med Sci Monit.

[7] Jones AE, Summers RL, Deschamp C, Galli RL (2001). A national survey of the air medical transport of high-risk obstetric patients. Air Med J.

[8] Gurshawn T, Jackson M, Barr J (2020). Transportation management affecting outcomes of patients with spontaneous intracranial hemorrhage. Air Med J.

[9] Tran QK, Famuyiwa O, Haase DJ (2020). Care intensity during transport to the critical care resuscitation unit: transport clinician's role. Air Med J.

[10] Bursac Z, Gauss CH, Williams DK, Hosmer DW (2008). Purposeful selection of variables in logistic regression. Source Code Biol Med.

[11] Rose M, Newton C, Boualam B (2019). Ground same intratransport efficacy as air for acute aortic diseases. Air Med J.

[12] Knobloch K, Dehn I, Khaladj N, Hagl C, Vogt PM, Haverich A (2009). HEMS vs. EMS transfer for acute aortic dissection type A. Air Med J.

[13] Bai G, Chanmugam A, Suslow VY, Anderson GF (2019). Air ambulances with sky-high charges. Health Aff (Millwood).

[14] Kelly M (2020). Sky-high air ambulance prices. Ann Emerg Med.

[15] Elliott JP, Trujillo R (1987). Fetal monitoring during emergency obstetric transport. Am J Obstet Gynecol.

[16] Poulton TJ, Gutierrez PJ (1992). Fetal monitoring during air medical transport. J Air Med Transp.

[17] Naruse K, Yamashita T, Onishi Y (2020). High-quality transmission of cardiotocogram and fetal information using a 5G system: pilot experiment. JMIR Med Inform.

